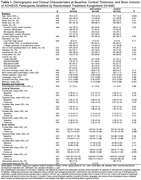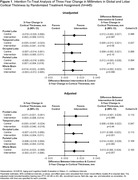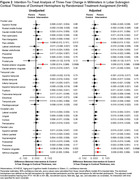# Effect of hearing intervention on three‐year change in brain morphology

**DOI:** 10.1002/alz.086740

**Published:** 2025-01-03

**Authors:** James Russell Pike, Alison R Huang, Jennifer A. Deal, Nicholas S Reed, Sheila Burgard, Theresa Chisolm, David Couper, Nancy W. Glynn, Theresa Gmelin, Adele M Goman, Lisa Gravens‐Mueller, Kathleen M. Hayden, Christine Mitchell, James Pankow, Victoria A Sanchez, Jennifer A Schrack, Marilyn S. Albert, Clifford R. Jack, David S. Knopman, Josef Coresh, Frank R Lin, ACHIEVE Collaborative Research Group

**Affiliations:** ^1^ New York University, New York, NY USA; ^2^ Johns Hopkins Bloomberg School of Public Health, Baltimore, MD USA; ^3^ University of North Carolina, Chapel Hill, NC USA; ^4^ University of South Florida, Tampa, FL USA; ^5^ University of Pittsburgh, Pittsburgh, PA USA; ^6^ Napier University, Edinburgh, Scotland United Kingdom; ^7^ Wake Forest University School of Medicine, Winston Salem, NC USA; ^8^ University of Minnesota, Minneapolis, MN USA; ^9^ Johns Hopkins University, Baltimore, MD USA; ^10^ Mayo Clinic, Rochester, MN USA

## Abstract

**Background:**

Prior longitudinal studies among older adults have documented associations between hearing loss and changes in brain morphology. Whether interventions involving hearing aids can reduce age‐related atrophy is unknown. A substudy within the Aging and Cognitive Health Evaluation in Elders (ACHIEVE, Clinicaltrials.gov Identifier: NCT03243422) randomized controlled trial tested the effect of a best‐practices hearing intervention versus health education control on three‐year change in cortical thickness among older adults with hearing loss.

**Method:**

The ACHIEVE study enrolled 977 community‐dwelling adults aged 70‐84 years at baseline (2018‐2019) with untreated hearing loss (better ear pure tone average [0.5‐4 kHz] ≥30 and <70 dB HL) and without substantial cognitive impairment from four sites across the U.S. (Jackson, MS, Forsyth County, NC, Minneapolis, MN, Washington County, MD). Participants were randomized to a hearing intervention (provision of hearing aids and related technologies, counseling, and education) or a health education control (individual sessions with a health educator covering topics relevant to chronic disease and disability prevention). Three‐dimensional magnetic resonance imaging was performed on 3 Tesla Siemens scanners in a subsample of 445 participants at the ACHIEVE baseline and three‐year follow‐up. Linear mixed effects models were used in intention‐to‐treat analyses to estimate three‐year change in cortical thickness. All models adjusted for baseline measures of hearing loss, recruitment source, site, age, sex, and education. Missing outcome and covariate data was imputed to mitigate bias caused by informative attrition.

**Result:**

At baseline, 224 participants were women (50.3%), 52 participants were Black (11.7%), and the mean (SD) age was 76.4 (4.0) years old (Table 1). Compared to the health education control, the hearing intervention exhibited a nominally protective effect on three‐year change in average cortical thickness (Figure 1). The greatest effect size for cortical thickness was observed in the occipital lobe, while the smallest effect size was detected in the temporal lobe. Statistically significant effects were detected in the pars orbitalis, rostral anterior cingulate, posterior cingulate, and isthmus cingulate (Figure 2).

**Conclusion:**

Hearing aid use may reduce decline in cortical thickness among older adults. The effects of hearing aids may be greatest in regions other than those associated with the auditory cortex.